# Supplementary material for: The Association between Smoking and Mortality in Women with Breast Cancer: A Real-World Database Analysis
Source: Cancers (Basel). 2022 Sep 20;14(19):4565. doi: 10.3390/cancers14194565 (PMC9558950; doi:10.3390/cancers14194565)
Supplement: Supplementary file 1 [file cancers-14-04565-s001.zip › cancers-1853192-supplementary.pdf]

Table S1. ICD-9 and ICD-10 Codes for Comorbidities

Comorbidities	ICD-9	ICD-10
**Myocardial infarction**	410, 412	I21, I22, I25.2
**Congestive heart failure**	398.91, 402.01, 402.11, 402.91, 404.01, 404.03, 404.11, 404.13, 404.91, 404.93, 428, 425.4-425.9	I09.9, I11.0, I25.5, I13.0, I13.2, I42.0, I42.5-I42.9, I43, I50, P29.0
**Peripheral vascular disease**	093.0, 437.3, 443, 441, 443.1-443.9, 471, 557.1	I70, I71, I73.1, I73.8, I73.9, I77.1, I79.0, I79.2, K55.1, K55.8, K55.9, Z95.8, Z95.9
**Cerebrovascular disease**	430-438, 362.34	G45, G46, I60-I69, H34.0
**Dementia**	290, 294.1, 331.2	F00-F03, F05.1, G30, G31.1
**Chronic pulmonary disease**	416.8, 416.9, 490-505, 506.4, 508.1, 508.8	I27.8, I27.9, J40-J47, J60-J67, J68.4, J70.1, J70.3
**Renal disease**	403.01, 403.11, 403.91, 404.02, 404.03 404.12, 404.13, 404.92, 404.93, 582, 583.0-583.7, 585, 586, 588.0	I12.0, I13.1, N03.2-N03.7, N05.2-N05.7, N18, N19, N25.0, Z49.0-Z49.2, Z94.0, Z99.2
**Hypertension**	401-405	I10-I13, I15
**Hyperlipidemia**	272	E78
**Diabetes**	250	E08-E13
**Liver disease**	070.6, 070.9, 070.22, 070.23, 070.32, 070.33, 070.44, 070.54, 456.0-456.2, 570, 571, 572.2-572.8, 573.3, 573.4, 573.8, 573.9	B18, I85.0, I85.9, I86.4, I98.2, K70.0-K70.4, K70.9, K71.1, K71.3-K71.5, K71.7, K72.1, K72.9, K73, K74, K76.0, K76.2-K76.9, Z94.4
